# Supplementary material for: Novel Treatments of Uveal Melanoma Identified with a Synthetic Lethal CRISPR/Cas9 Screen
Source: Cancers (Basel). 2022 Jun 29;14(13):3186. doi: 10.3390/cancers14133186 (PMC9264875; doi:10.3390/cancers14133186)

Blots Figure 1B

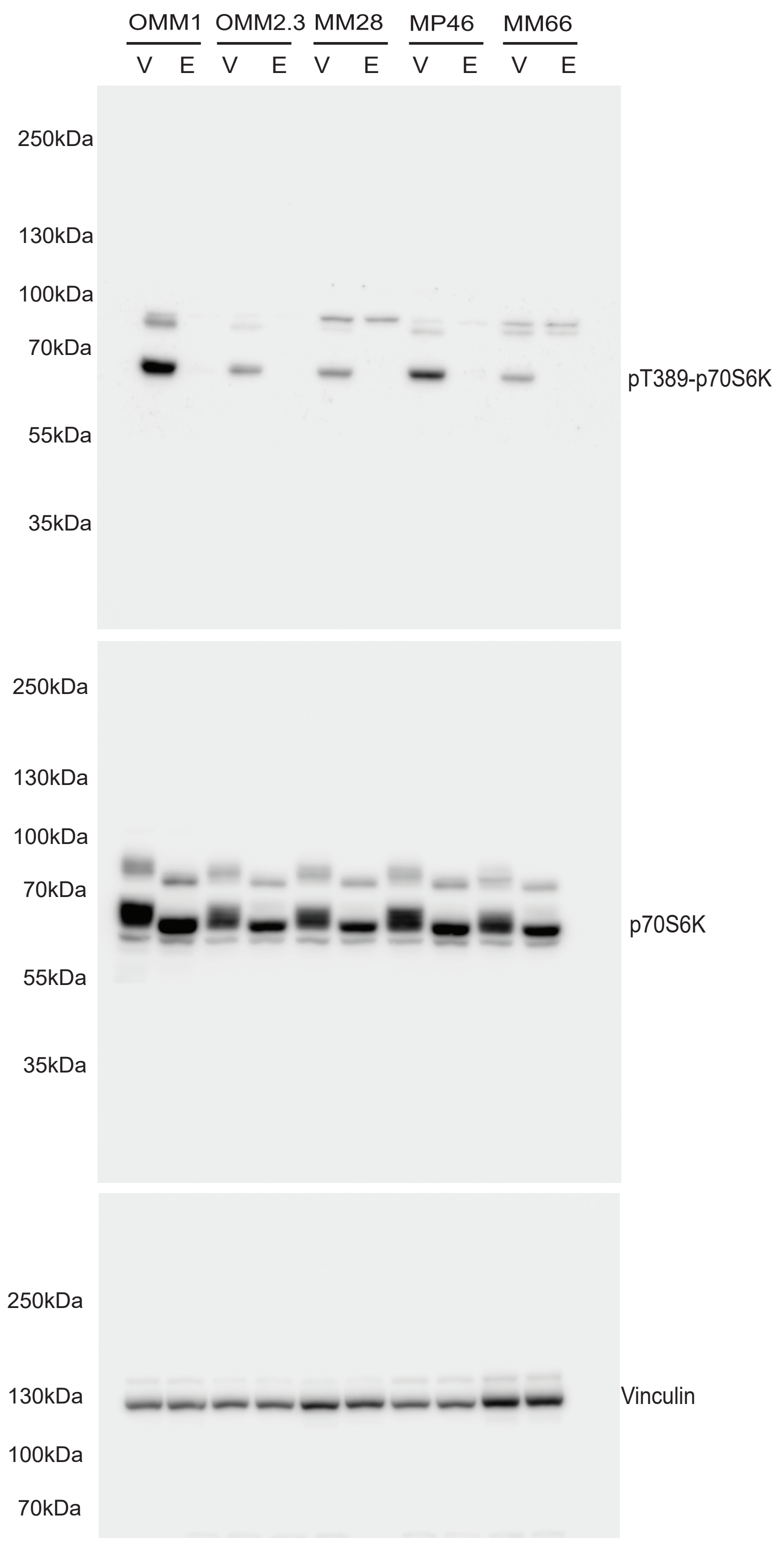

Blots Figure 4A

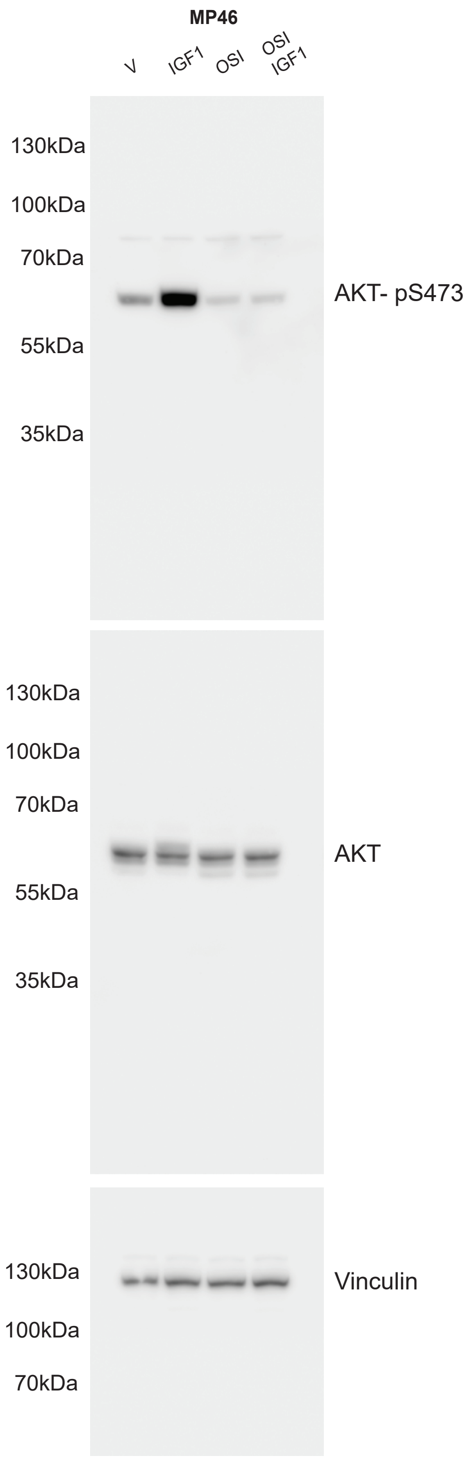

Blots Figure 4B

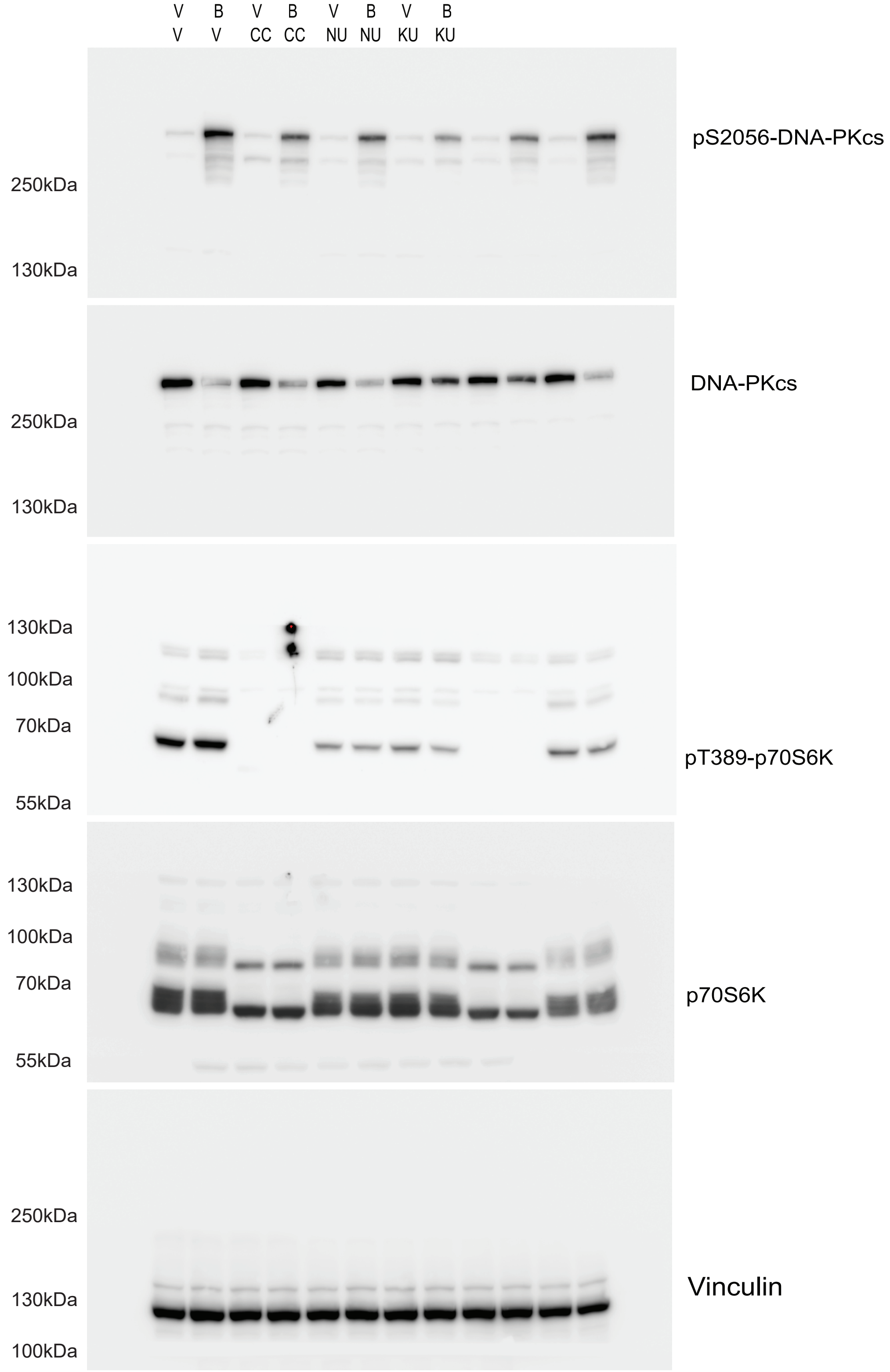

Blots Figure 6A

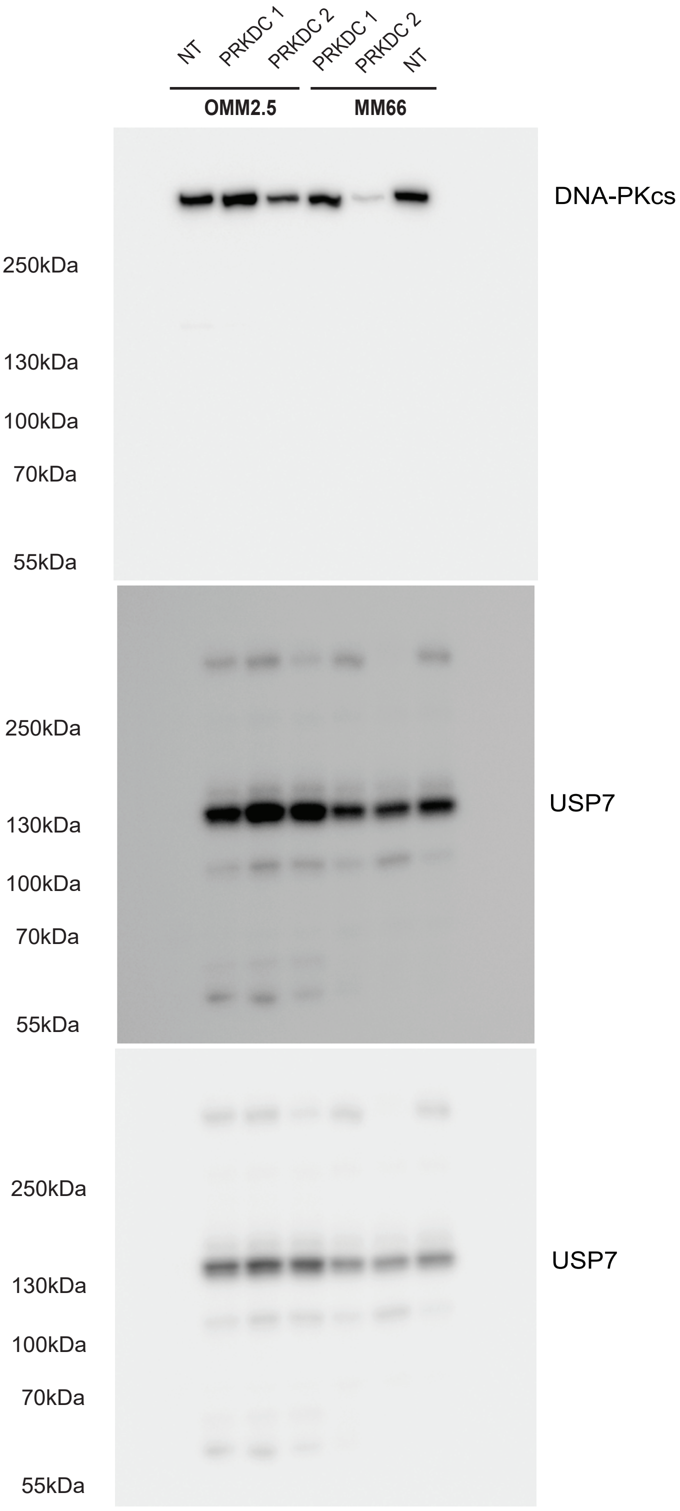

Blots Figure 6B

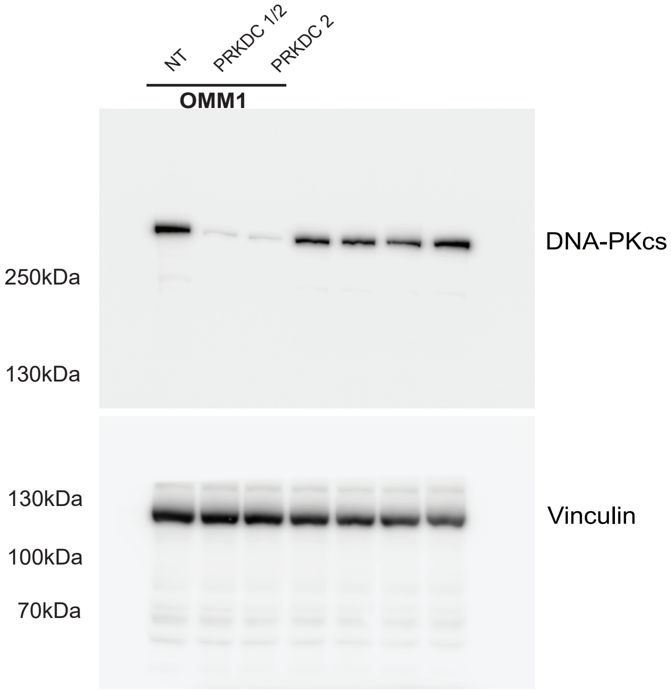

Blots Figure 6C

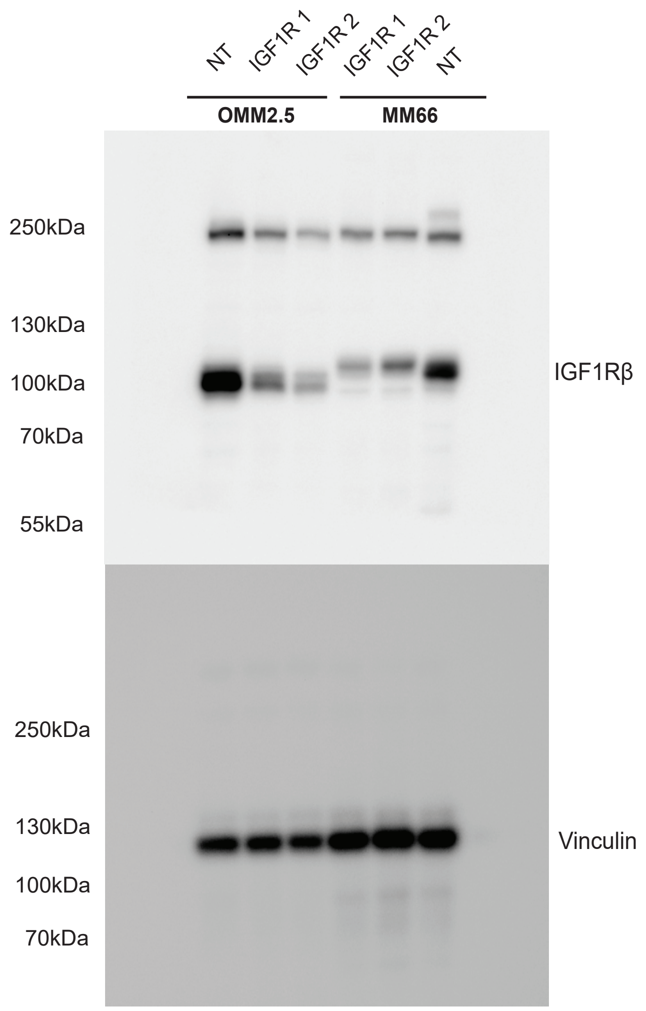

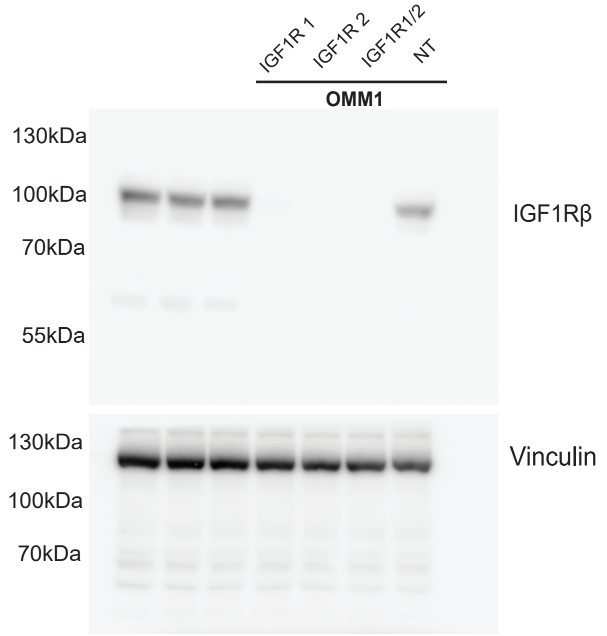

Blots Figure S3 D

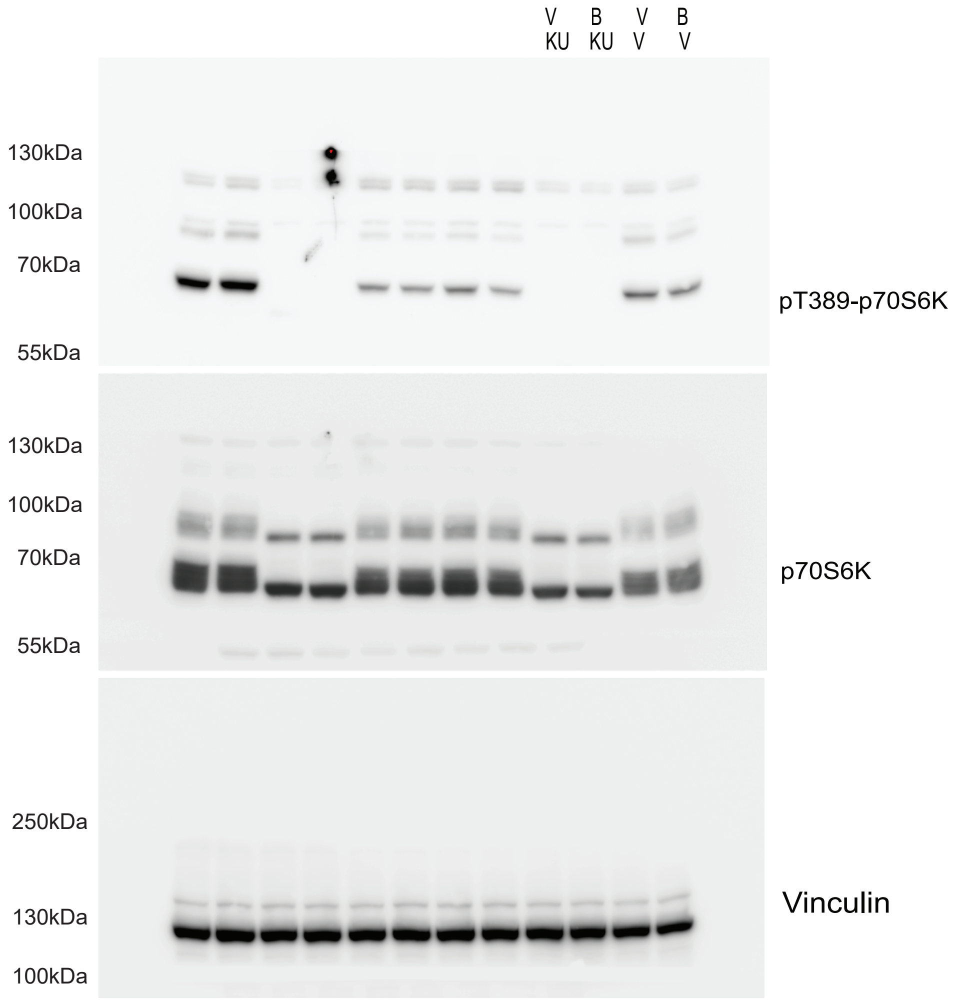

Supplement: Supplementary file 1 [file cancers-14-03186-s001.zip › Supplementary Figure S5.pdf]
