# Supplementary material for: Novel Treatments of Uveal Melanoma Identified with a Synthetic Lethal CRISPR/Cas9 Screen
Source: Cancers (Basel). 2022 Jun 29;14(13):3186. doi: 10.3390/cancers14133186 (PMC9264875; doi:10.3390/cancers14133186)
Supplement: Supplementary file 1 [file cancers-14-03186-s001.zip › Supplementary Figures.pdf]

**Figure S1**

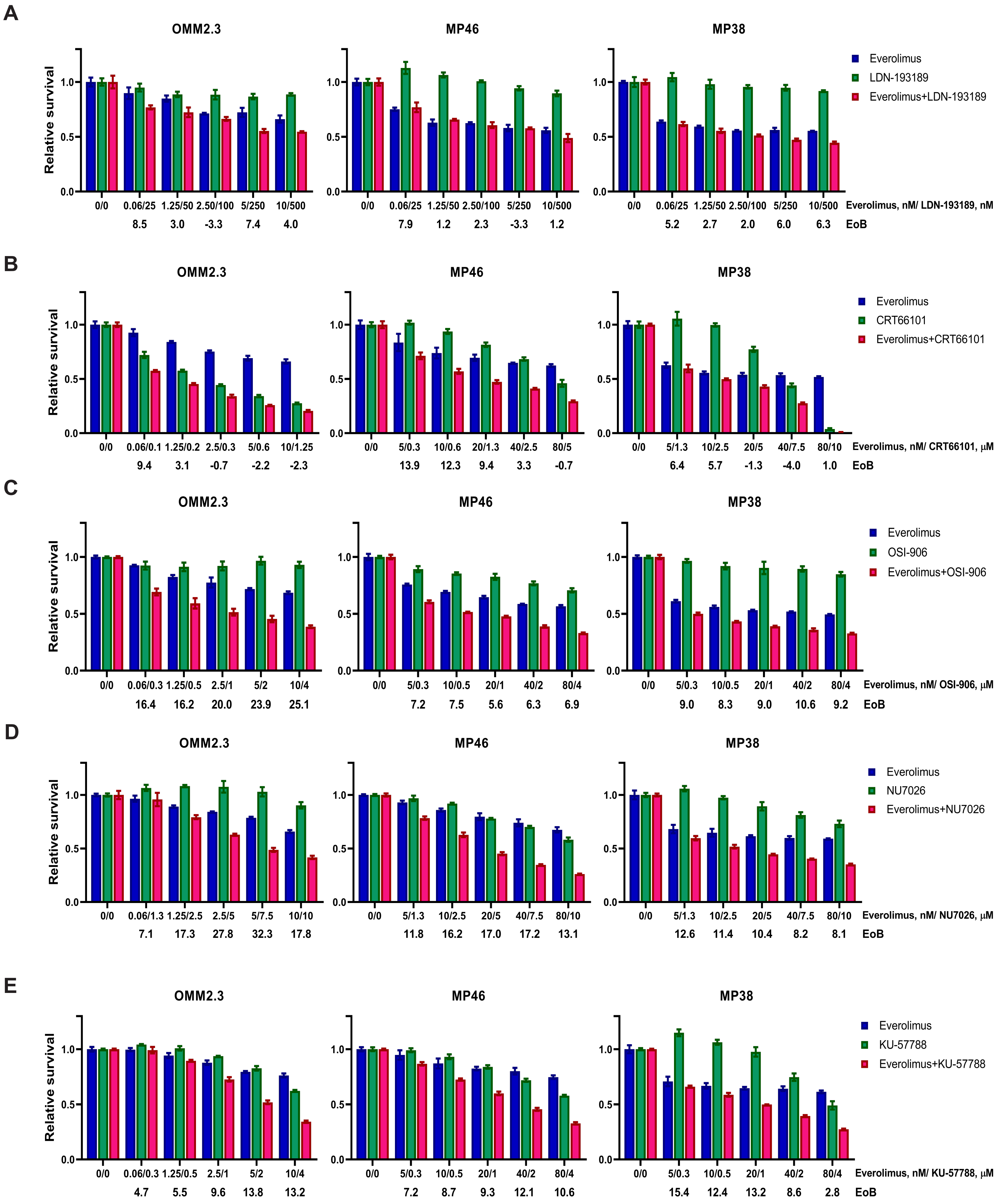


Figure S1: Synergism of everolimus in combination with targeted inhibitors of DNA-PKcs or IGF1R.
Uveal melanoma cell lines OMM2.3, MP46 and MP38 were treated with either everolimus (blue bars),or a targeted inhibitor of (A) ALK-2 (LDN-193189,) (B) PRKD3 (CRT66101), (C) IGF1R (OSI-906), (D) DNA-PKcs (NU7026),
(E) DNA-PKcs (KU57788) (green bars), or the combination of both (red) for 5 days to determine the effect on the cell viability.
The synergistic scores were calculated by Excess over Bliss algorithm;
they are indicated for each pair of concentrations below the X-axes (EoB);

**Figure S2**

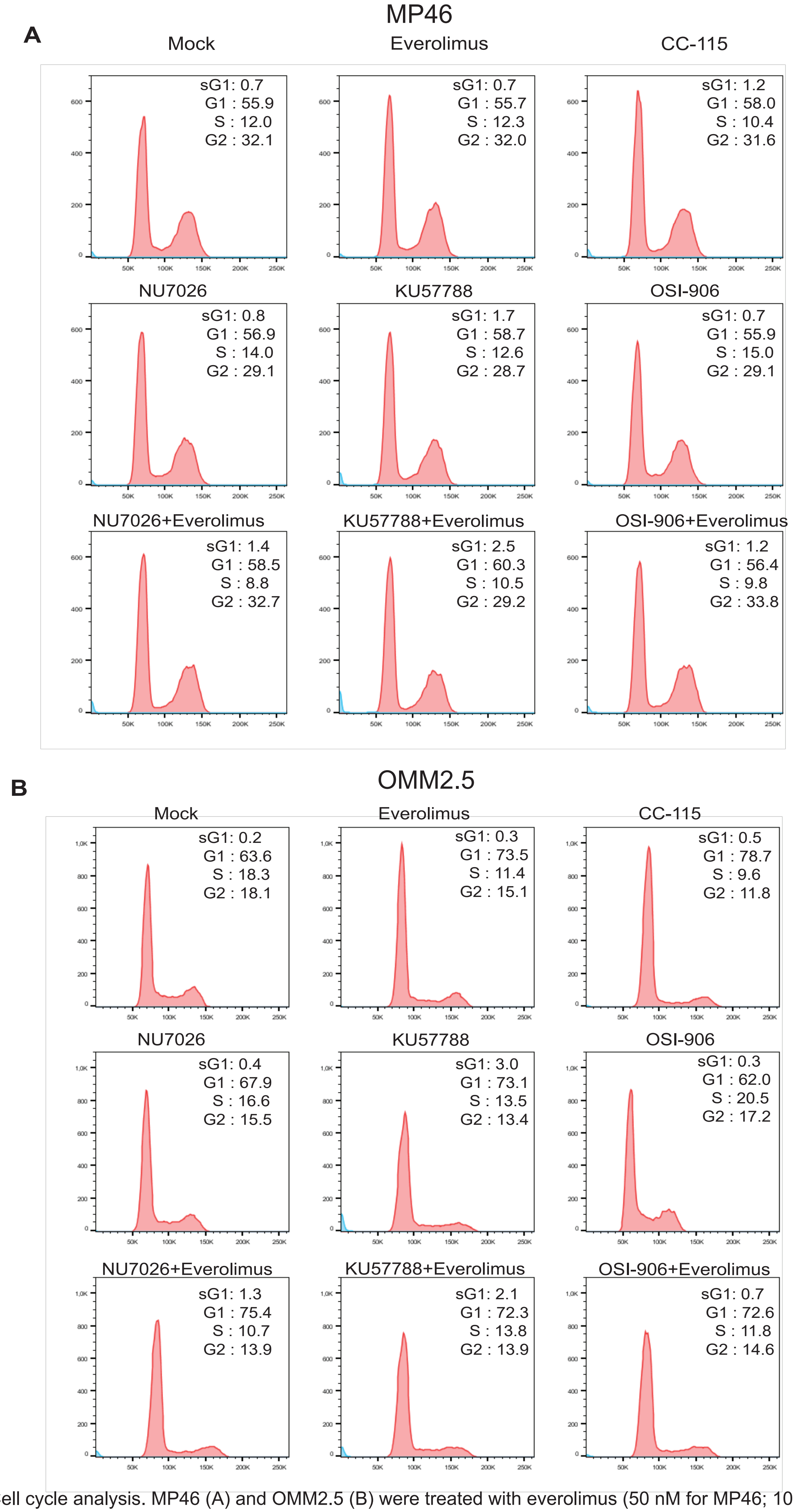


Figure S2: Cell cycle analysis. MP46 (A) and OMM2.5 (B) were treated with everolimus (50 nM for MP46; 10 nM for OMM2.5) alone or in combination with NU7036 (10 µM), or KU57788 (2 µM), or OSI-906 (3 µM), or with CC-115 (300 nM) for 5 days.

## Figure S3

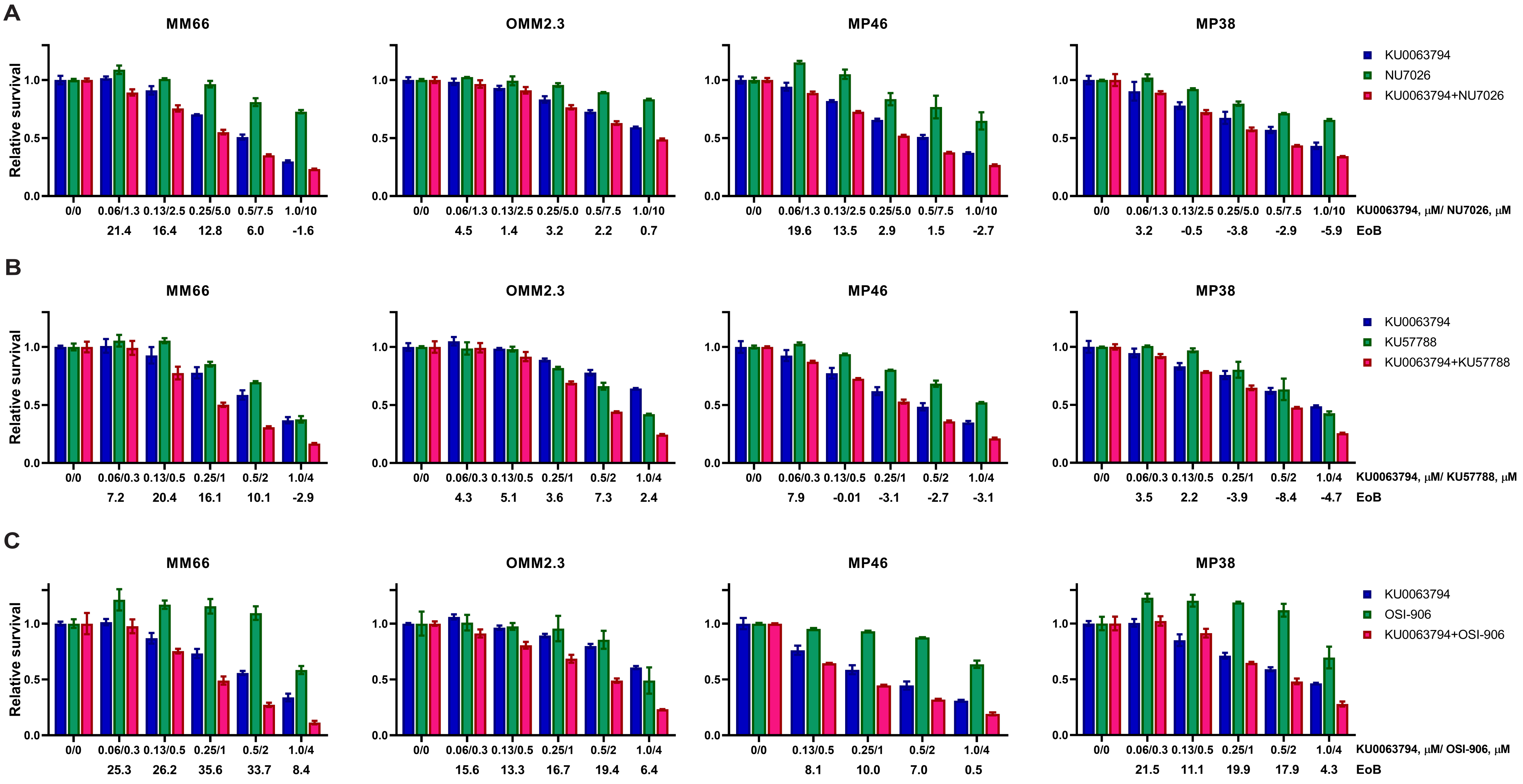


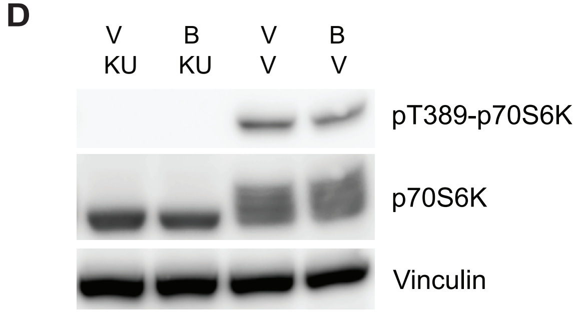


Figure S3: Synergism of KU0063794 in combination with DNA-PKcs or IGF1R inhibitors.(A-C) Uveal melanoma cell lines OMM2.3, MP46, MP38 were treated with either KU0063794 (blue bars), or a targeted inhibitor of (A) DNA-PK (NU7026), (B) DNA-PK (KU57788), (C) IGF1R (OSI-906) (green bars), or the combination of both (red) for 5 days to determine the effect on the cell via-bility. The synergistic scores were calculated by Excess over Bliss algorithm; they are indicated for each pair of concentrations below the X-axes (EoB). (D) MP46 were pre-treated with either vehicle (V) or 1 μM KU0063794 for 2 hours. After addition of bleomycin (B) the cells were incubated for 4 hours and harvested for analysis.

# Figure S4

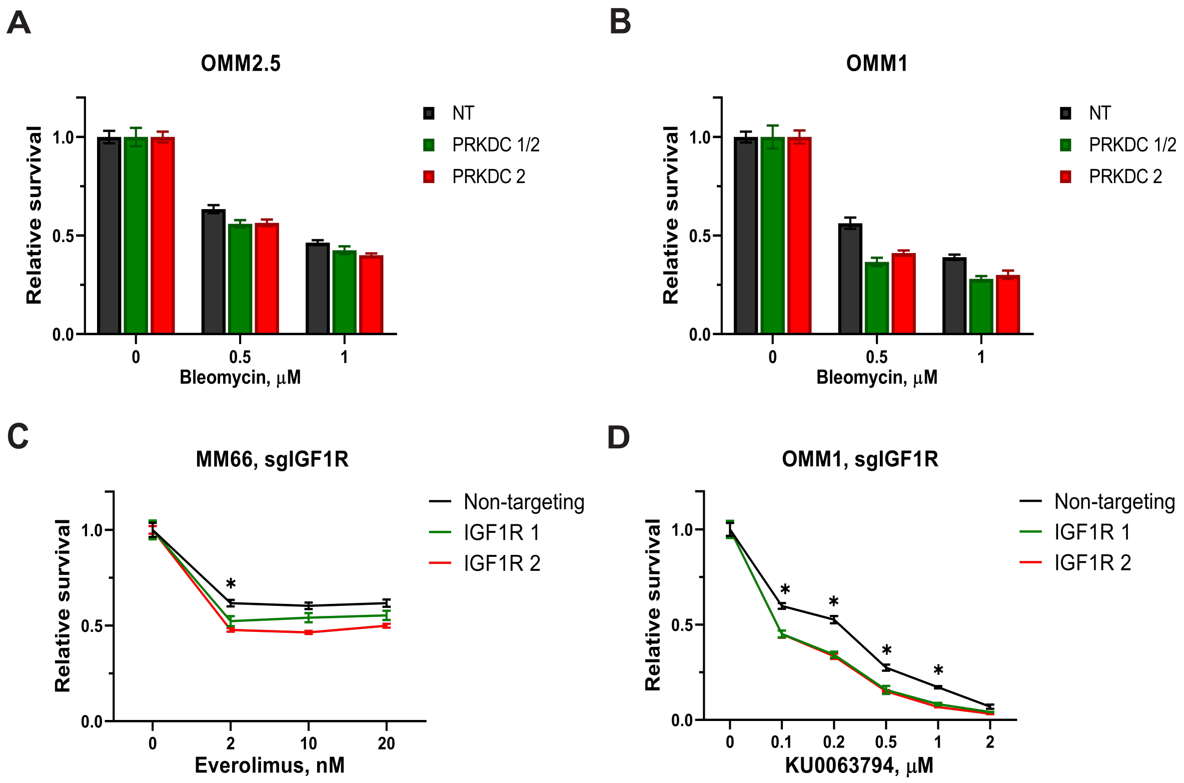


Figure S4: Genetic verification of the hits. (A-B) Effect of combination of bleomycin with CRISPR/Cas9-mediated knockout of PRKDC on viability of OMM2.5 (A) and OMM1 (B) after 5 days of treatment. (C) Effect of combination of everolimus with depletion of IGF1R on viability of MM66 cells after 4 days of treatment. (D) Effect of combination of KU0063794 with depletion of IGF1R on viability of OMM1 cells after 5 days of treatment. Asterisk (*) indicates significant differences (p<0.05) between Non-targeting control and both sgRNAs.
